# Relationship Between Muscular Activity and Assistance Magnitude for a Myoelectric Model Based Controlled Exosuit

**DOI:** 10.3389/frobt.2020.595844

**Published:** 2020-12-17

**Authors:** Francesco Missiroli, Nicola Lotti, Michele Xiloyannis, Lizeth H. Sloot, Robert Riener, Lorenzo Masia

**Affiliations:** ^1^Institut für Technische Informatik (ZITI), Heidelberg University, Heidelberg, Germany; ^2^Institute of Robotics and Intelligent Systems, ETH Zurich, Zurich, Switzerland; ^3^Spinal Cord Injury Center, Balgrist University Hospital, Medical Faculty, University of Zurich, Zurich, Switzerland

**Keywords:** soft exosuit, electromyography, inertial measurement units, human-robot interaction, kinematics, muscular fatigue

## Abstract

The growing field of soft wearable exosuits, is gradually gaining terrain and proposing new complementary solutions in assistive technology, with several advantages in terms of portability, kinematic transparency, ergonomics, and metabolic efficiency. Those are palatable benefits that can be exploited in several applications, ranging from strength and resistance augmentation in industrial scenarios, to assistance or rehabilitation for people with motor impairments. To be effective, however, an exosuit needs to synergistically work with the human and matching specific requirements in terms of both movements kinematics and dynamics: an accurate and timely intention-detection strategy is the paramount aspect which assume a fundamental importance for acceptance and usability of such technology. We previously proposed to tackle this challenge by means of a model-based myoelectric controller, treating the exosuit as an external muscular layer in parallel to the human biomechanics and as such, controlled by the same efferent motor commands of biological muscles. However, previous studies that used classical control methods, demonstrated that the level of device's intervention and effectiveness of task completion are not linearly related: therefore, using a newly implemented EMG-driven controller, we isolated and characterized the relationship between assistance magnitude and muscular benefits, with the goal to find a range of assistance which could make the controller versatile for both dynamic and static tasks. Ten healthy participants performed the experiment resembling functional daily activities living in separate assistance conditions: without the device's active support and with different levels of intervention by the exosuit. Higher assistance levels resulted in larger reductions in the activity of the muscles augmented by the suit actuation and a good performance in motion accuracy, despite involving a decrease of the movement velocities, with respect to the no assistance condition. Moreover, increasing torque magnitude by the exosuit resulted in a significant reduction in the biological torque at the elbow joint and in a progressive effective delay in the onset of muscular fatigue. Thus, contrarily to classical force and proportional myoelectric schemes, the implementation of an opportunely tailored EMG-driven model based controller affords to naturally match user's intention detection and provide an assistance level working symbiotically with the human biomechanics.

## 1. Introduction

The relatively novel frontier of soft wearable robotics and exosuits provided several solutions and tools potentially impacting multiple realms: from supporting people with neurological disorders, to improving labor efficiency in industrial settings by augmenting human motor capabilities. The use of textiles and elastomers, intrinsically complying with the complex human biomechanics, has allowed a substantial leap in the rendering of Human-Robot interaction (HRI): explicitly in contrast with rigid exoskeletons, exosuits do not have the disadvantage of kinematic incompatibility with the human joints and they are designed with negligible distal mass on human limbs. These characteristics have allowed researchers and developers to reduce the energy cost of human walking and running (Kim et al., 2020) and support the upper limbs against gravity in both unimpaired users (Thalman et al., 2018) and neurological patients (O'Neill et al., 2020).

Exosuits are designed to follow and support human movements, working in parallel with the human muscles to apply, in part or fully, the forces required to complete a variety of tasks. Detecting the user's intention and correctly providing assistive force in a timely manner is thus the key aspect for the effectiveness and a wide acceptance of such technology. While the rhythmic nature of human walking has allowed to exploit stereotypical gait events to recognize human intentions (e.g., Grimmer et al., 2019), upper limb dexterity, and its large tasks manifold, present a much more challenging and open problem.

Intention detection strategies for exosuits that support the upper limbs are often limited to a manual input and open loop command, operated by an assistant or by the user her/himself: for example, for soft devices driven by a Pneumatic Interference Actuator (PIA) for shoulder support as described by O'Neill et al. (2020). A similar strategy was chosen by Simpson et al. (2020) for supporting shoulder abduction or by Thalman et al. (2018), to assist elbow flexion. While this method is extremely practical and robust, at the same time it lacks versatility and relies on an additional, functional “hand” which must intervene in order to control a contralateral assistive device.

A more versatile and effective approach is that of compensating for the intrinsic dynamics of the tasks, gathering information from force and kinematic sensors: Xiloyannis et al. (2019) used an indirect force controller to deliver a torque equal and opposite to gravity at the elbow; gravitational forces were estimated from a rotary encoder on the joint and successively closed in the control loop. This was done using a force sensor located in series with the suit's artificial tendon which was responsible of providing the actuation torque and therefore compensate the dynamics of the lifting task.

The downsides of this approach were two-fold: (1) the controller was not adaptive to changing external conditions, only compensating for the dynamics defined by its control laws (e.g., when the user picks up an object, different loads are assisted with the same torque) and (2) the controller depended on the detection of an internal interaction force to understand the user intention, introducing an inevitable delay between initiation of movement and exosuit assistance. A perceivable time lag between user motion and hardware action deeply jeopardizes the intuitiveness of control and decrease the chance of having an effective embodiment between the wearer's body and the device.

To bridge these two gaps, in Lotti et al. (2020) we tapped into the efferent motor signals from the human central nervous system (CNS). Surface electromyography (sEMG) precedes the mechanical manifestation of movement and carries information on the CNS adaption to changing external dynamics. Using a subject-specific, real-time and physically-accurate model of the muscle-tendon geometry and dynamics, we achieved high accuracy in estimation of joint torques (≤0.02 Nm/kg) with an intervention delay from the exosuit within the physiological electromechanical time lag between EMG onset and muscle force generation (≤55 ms). For a variety of tasks, this resulted in a reduction of the activation of the major muscles responsible for flexing the elbow of up to 73%.

The encouraging results found in Lotti et al. (2020) led us to further investigate the effect of a myoelectric-controlled exosuit on the biomechanics of human arm movements. In particular, the objective of the current contribution is to isolate and characterize the relationship between assistance magnitude and muscular benefits for the user. This is not a trivial mapping, as EMG activity is both the driving signal and the primary index of the effectiveness of assistance. Moreover, in the proposed EMG-driven model based controller, a minimal level of muscular activity will always be required to drive exosuit assistance: increasing assistance gain will likely result in higher delivered torques from the robot but will also amplify noise from the EMG signals. Besides that, high assistive gains might result in uncontrolled jerky movements, with a consequent increase in the antagonistic muscular activity, and a lack of synchronization between the biological and artificial actions. All these aspects hint to the existence of an optimal point for the exosuit intervention, corresponding to a trade-off between reduction in muscular activity and quality of assistance.

Due to the adaptive nature of the aforementioned controller, we hypothesize that increasing the assistance will lead to an increasingly higher reduction in the activation of the major muscles working in parallel with the robot. As a secondary hypothesis speculated that increasing levels of exosuit assistance will result in movements lacking of smoothness, in line with what observed in our previous work (Xiloyannis et al., 2019). Understanding the relationship underlaying human motor intention and robotic intervention, is paramount for exploiting an effective assistive technology: hence, the current study aimed at providing experimental evidences that, an opportunely implemented control strategy using biosignals in the loop, can be a good candidate to efficiently regulate the interaction between the wearable device and its user with potentials future applications in both industrial settings and rehabilitation environments.

## 2. Materials and Methods

### 2.1. Exosuit Design

The real-time EMG-driven model based controller runs on a novel version of the upper-limb soft wearable exosuit presented in Xiloyannis et al. (2019), and designed to assist elbow flexion/extension.

**Figure 1:**
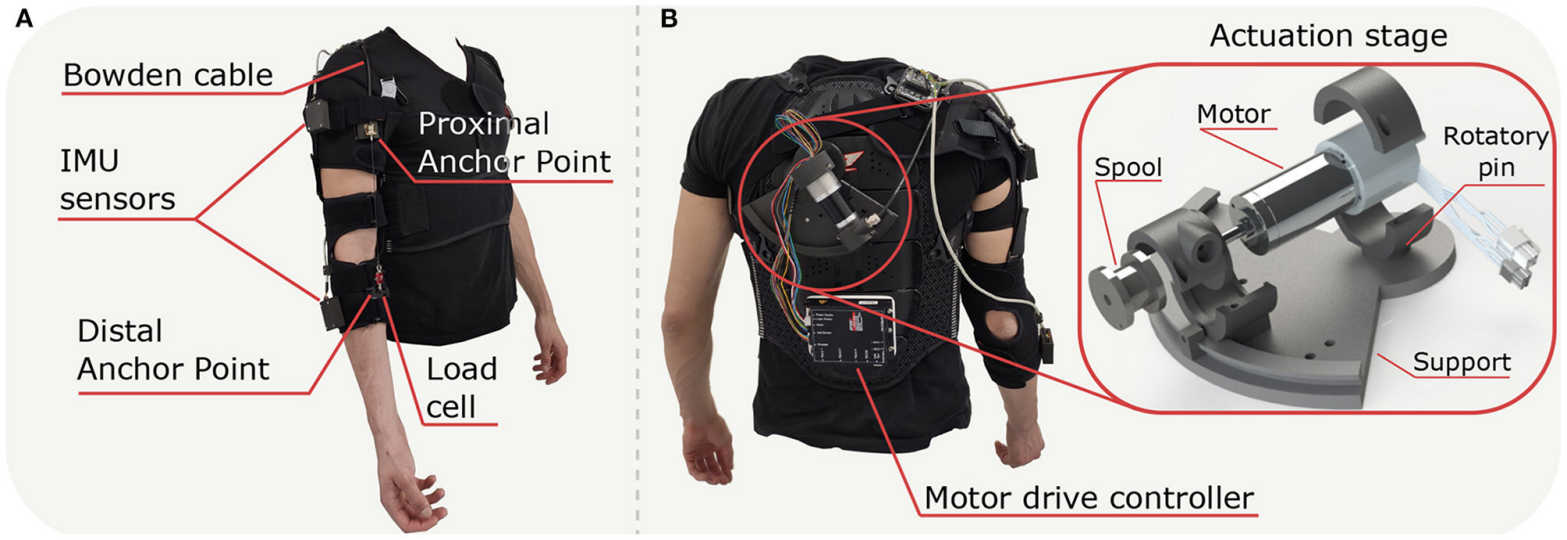
*Exosuit* developed to assist the elbow in flexion and extension; **(A)** frontal view of the apparatus with positioning of the principal components, **(B)** back view of the suit with focus on the *Actuation stage* and connection of the *Motor drive controller*.

The exosuit's textile frame uses a customized passive orthosis (Sporlastic- NEURO-LUX II, Nürtingen, Germany) and it comprises of two soft fabric components connected by straps and wrapped around shoulder, upper arm and forearm (Figure 1A). We designed a customized support for the actuation stage and the motor drive controller (EPOS2 50/5, Maxon, Sachseln, Switzerland), placed on a customized back protector (Zandonà - Evo X6, Caerano di San Marco, TV, Italy). The actuation stage has been designed as a 3D printed tilting system (Markforge, Watertown MA, USA) able to rotate around a fixed pin to adapt to the subjects' sizes (Figure 1B).

The exosuit transmits assistance to the wearer through an artificial tendon, routed in a Bowden sheath from the motor to the elbow joint. The whole tendon-driven actuation unit includes the following components: (1) a brushless DC motor (Maxon- EC-i 40, 70 W, Sachseln, Switzerland); (2) planetary gearhead (Maxon- GP32C, ø32 mm 51:1, Sachseln, Switzerland) in series with the motor; (3) a spool (ø22 mm) coupled to the gear-head shaft around which a tendon is wound; and (4) an incremental encoder (Maxon-ENC 16 EASY XT, 1024 pulses/rev, Sachseln, Switzerland) to measure the angular position.

The Bowden cable cover (Shimano SLR, ø5 mm, Sakai, Õsaka, Japan) was attached to the upper arm strap, while its inner tendon (Black Braided Kevlar Fiber, KT5703-06, 2.2 kN max load, Loma Linda CA, USA) to the load cell, anchored to the forearm strap. The motor transfers the power to the anchor points located on the proximal and distal sides of the elbow joint and the assistance is delivered by applying a pulling force to the cable. When the motor tensions the artificial tendon, it pulls the two anchor points together, applying a flexing moment on the elbow joint. The distal anchor point was made by an Onyx component 3D printed and sewed on the orthosis; a load cell (Futek, FSH04416, Irvine CA, USA), tied to the distal anchor point with Kevlar fiber, measures the tension in the flexing tendon. We mounted two Inertial Measurement Units (IMUs) (Bosch, BNO055, Gerlingen, Germany) on the arm and forearm, fixed to the orthosis by velcro straps, to record the angular position at the elbow.

The suit could be easily donned and doffed using velcro straps and a cuff lacing system, allowing the suit to adapt to users of different sizes. The major advantages of this new design compared to our previous prototype (Lotti et al., 2020) are: (1) the removal of any rigid component at the elbow joint to measure angular displacement; (2) the placement of the anchor points further from the elbow axis of rotation, allowing the device to cover the entire physiological joint range of motion; (3) the variable orientation for the actuation stage support, placed on the back protector which reduces the overall volume of the hardware.

### 2.2. Real-Time Control Framework

**Figure 2:**
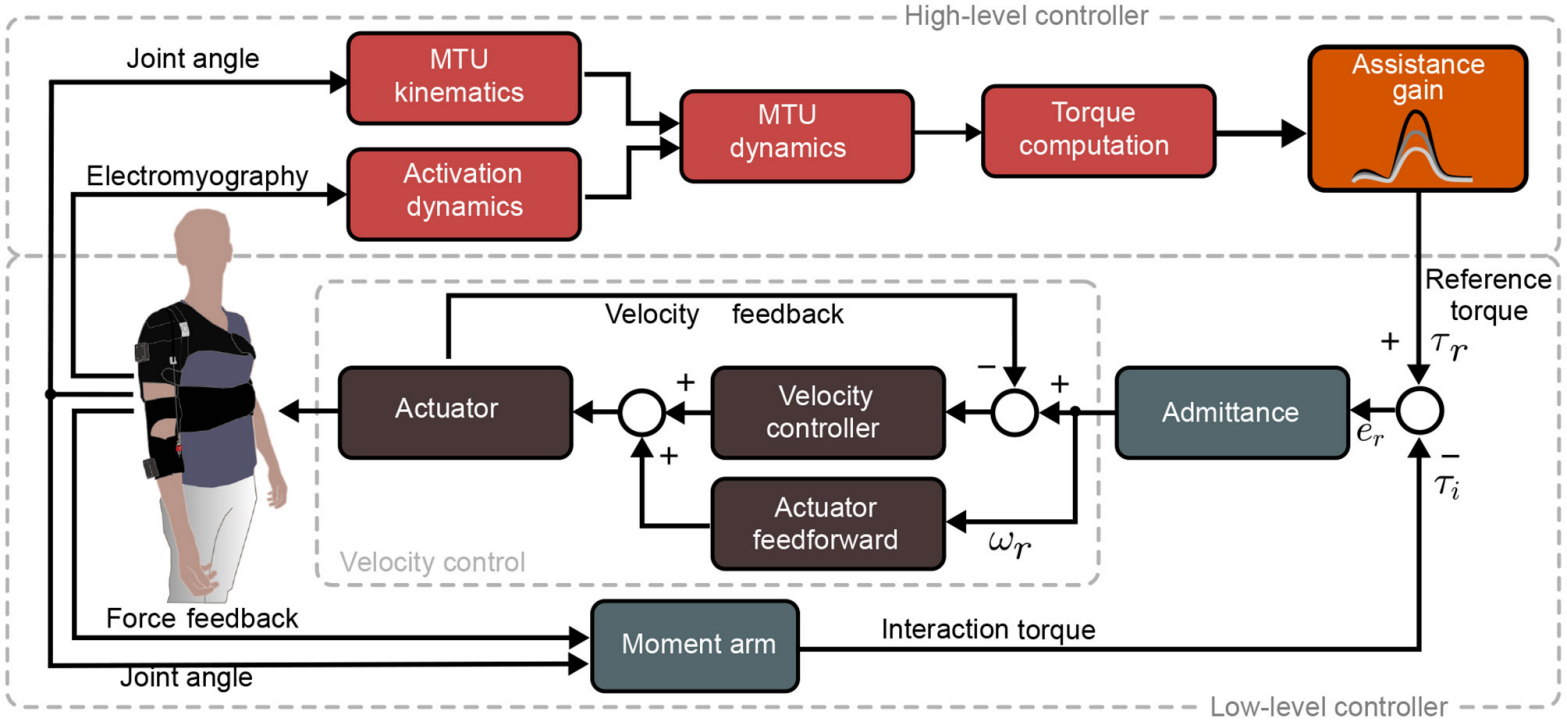
Real-time control framework: The *High level controller* (red) estimates physiologically accurate torque profiles equal to the torque generated by the muscles acting on the elbow joint (τ_*r*_). This is then tracked by an admittance controller comprised of an outer torque loop (light gray) and an inner velocity loop (dark gray). The estimated torque is compared to the torque (τ_*i*_) delivered by the exosuit to the human joint. The error torque (*e*_*r*_) is then converted to a motor velocity, delivering assistive power to the user through bowden cable.

The exosuit assistive torque was evaluated by an EMG-driven control paradigm (Figure 2). The architecture included two layers: a high-level controller estimated the desired torque at the elbow level, starting from EMG signals and kinematic data, and a low-level controller for torque tracking which directly commands the tendon-driven electromechanical stage. The latter was achieved through an admittance controller, with an internal velocity loop, closed on the motor encoder.

#### 2.2.1. High-Level Controller: Myoprocessor Module

The myoprocessor module has been adapted from the implementation proposed in Lotti et al. (2020). It estimates the torque generated at the elbow for flexion/extension, merging the EMG signals of the main muscles that span the joint (i.e., the long head of biceps and the long head of the triceps) and the kinematic data extracted from the IMUs. The module comprises four interconnected blocks with specific functions as illustrated in Figure 2 and explained in the details in the following sections.

##### 2.2.1.1. Activation Dynamics

The activation dynamic block converts the EMG signal envelope of the *j*-muscle *u*_*j*_ into muscle activation *a*_*j*_ through a non-linear transfer function based on (Lloyd and Besier, 2003):

(1) $$\begin{array}{l} a_{j}\left(t\right)=\frac{e^{A_{j}u_{j}\left(t\right)}-1}{e^{A_{j}}-1}. \end{array}$$

##### 2.2.1.2. MTU Kinematics

The Musculo Tendon Unit kinematic block is responsible to extract the three-dimensional arm musculoskeletal geometry (i.e., muscle tendon length and moment arm) by means of a set of multidimensional cubic B-splines (Sartori et al., 2012) starting from subject specific and opportunely scaled Opensim models (Holzbaur et al., 2005).

##### 2.2.1.3. MTU Dynamics

This unit takes as input the *Activation dynamics* and *MTU kinematics* outputs and models the muscles contraction force. The force $F_{j}^{MT}\left(t\right)$ produced by the *j*-muscle was obtained by the relationship:

(2) $$\begin{array}{l} F_{j}^{MT}\left(t\right)=F_{j}^{max}\left[a_{j}\left(t\right)f_{l}\left(t\right)f_{v}\left(t\right)+f_{p}\left(t\right)\right]\cdot \cos\phi_{j}\left(t\right) \end{array}$$

where $F_{j}^{max}$ is the maximum isometric force (Zajac, 1989), *a*_*j*_(*t*) the muscle activation, *f*_*l*_, *f*_*v*_, and *f*_*p*_ are, respectively, the force-length relationship, the force-velocity relationship and the parallel passive elastic muscle force. Finally, ϕ_*j*_ is the pennation angle of the fibers. The force-length (*f*_*l*_) and the force-velocity (*f*_*v*_) relationships represent the contractile element of the muscle. The first one is modeled via a Gaussian function that describes the dependence of the steady-state isometric force of a muscle as a function of muscle length (Winters, 1990). The force-velocity relationship (*f*_*v*_) is the dynamic response of the muscle fibers during the contraction (Katz, 1939). The parallel passive elastic muscle force (*f*_*p*_) describes the passive element of the muscle and is achieved from an exponential relationship, which allows to obtain the passive forces regardless of fiber length, thus accounting for non-zero passive forces (Schutte et al., 1993). As simplifying assumption, we consider the tendon as non-deformable, i.e., with a constant length $l^{t}=l_{s}^{t}$.

##### 2.2.1.4. Torque Computation

The last block combines the muscle forces *F*^*MT*^(*t*) and the muscle moment arm vector *J*(*t*) to obtain the torque at the joint level τ_*r*_(*t*) as follows:

(3) $$\begin{array}{l} \tau_{r}\left(t\right)=J{\left(t\right)}^{T}\cdot F^{MT}\left(t\right) \end{array}$$

#### 2.2.2. Low-Level Controller

We used the output torque from the high-level controller as desired input to the exosuit low-level admittance controller. It comprises two nested loops: a feedback torque and an inner feedback + feedforward velocity loop. The torque loop compares the reference torque, τ_*r*_, to the interaction torque τ_*i*_ extracted by the load cell located at the distal anchor point to record the cable tension (*f*):

(4) $$\begin{array}{l} \tau_{i}=J{\left(\phi_{e}\right)}^{T}f \end{array}$$

*J*(ϕ_*e*_) is the artificial tendon moment arm of the suit respect to the elbow joint along the axis of rotation, namely the sagittal plane. This value is function of the elbow angle ϕ_*e*_ and was defined as follows:

(5) $$\begin{array}{l} J\left(\phi_{e}\right)=\frac{\partial h_{f}}{\partial \phi_{e}} \end{array}$$

where *h*_*f*_(ϕ_*e*_) is the function describing the cable displacement as function of geometry and joint angle:

(6) $$\begin{array}{l} h_{f}\left(\phi_{e}\right)=\left(2\sqrt{a^{2}+b^{2}}\right)\cdot \cos\left(\tan^{-1}\left(\frac{a}{b}\right)+\frac{\phi_{e}}{2}\right)-2b \end{array}$$

*a* and *b* are, respectively, the half-width of the forearm and the distance between the elbow flexion point and the distal anchor point.

To transform the tracking torque error *e*_*r*_ = τ_*r*_ − τ_*i*_ into the desired angular velocity, ω_*r*_ we used a PID admittance controller with transfer function:

(7) $$\begin{array}{l} Y\left(s\right)=\frac{\omega_{r}}{e_{r}}=\frac{K_{p}+K_{i}\cdot \frac{1}{s}}{1+K_{d}\cdot s} \end{array}$$

where the gains *K*_*p*_, *K*_*i*_ and *K*_*d*_ shape the dynamic response of the exosuit in order to follow the estimated torque τ_*r*_ as accurate as possible minimizing time response and settling time by the target *e*_*r*_. The role of the velocity loop consisted of a compensation of the intrinsic, unwanted dynamics of the exosuit (i.e., backlash, Coulomb, and viscous friction) and it was experimentally tuned to have a stiff, slightly underdamped response. Feedforward acceleration and velocity terms were added to the feedback, to improve the tracking accuracy and the bandwidth.

Gains were tuned on a single subject before starting the experimental procedure and their values were left unchanged for all the participants enrolled in the tests. We choose to use the hardware with a previously tuned low level controller in order to ensure the same dynamic behavior and assistance performance of the exosuit across all subject, therefore reducing inter-group variability.

#### 2.2.3. Myoprocessor Calibration

The high level controller (the myoprocessor module) needed to be opportunely tailored on each subject by means of calibration procedure.

The calibration pipeline has three different steps: (i) a maximum voluntary contraction (MVC) trial, (ii) a static pose measurement using a motion capture system, and (iii) a dynamic calibration recording users'arm motion. The MVC consisted on muscle isometric contraction, and was used to normalized the EMG signals and set the EMG gain values. The open-source software OpenSim allows to linearly scale a generic musculoskeletal model (Holzbaur et al., 2005) based on the markers position, to match participants' individual arm anthropometry.

The dynamic calibration required subjects to follow reference elbow trajectories, visually fed-back on a screen through a phantom dummy. At the beginning of the task, the phantom dummy kept the elbow fully extended (0°) for 20 s, to acquire baseline sEMG data at rest. After 20 s, the phantom dummy started to perform elbow movements with the following relationship:

(8) $$\begin{array}{l} \theta_{d}\left(t\right)=A_{0}+A\sin\left(2\pi f\left(t\right)t\right) \end{array}$$

with *A*_0_ = *A* = 45°, and *f* being a step-wise varying frequency in increasing steps of 0.05 Hz, between 0.05 and 0.5 Hz. These set values were chosen as they correspond to movements with a peak velocity between 12.5°/s and 150°/s, respectively, equivalent to 10 and 120% of the speed of the elbow in daily tasks (Buckley et al., 1996). Each frequency value was presented to the participants for 20 s.

Recorded EMG signals, joint angles and reference torques, acquired during the static and dynamic calibration, were used to tune the myoprocessor module. Through the *myoprocessor* calibration, we estimated the values of the internal model parameters that minimized the normalized error between the *myoprocessor* predicted torque and the reference torque profiles, extracted from the scaled musculoskeletal model by using the Opensim inverse dynamics toolkit. Tuned parameters included optimal fiber length, tendon slack length, maximal isometric force and EMG-to-activation filtering coefficients.

### 2.3. Experimental Setup

In order to test the exosuit performance, we used a multi-channel EMG system (Trigno wireless, Delsys, Natick MA, USA) and recorded six muscles involved in the upper limb movements: *Biceps, Triceps, Anterior Deltoid, Posterior Deltoid, Pectoralis, and Trapezius*. Electrodes placement followed the SENIAM guidelines (Hermens et al., 2000).

A DAQ board (Quanser QPIDe, Markham, Ontario, Canada) was used to acquire all signals, with a sampling frequency of 1 kHz. The real-time control and data logging was implemented in a MATLAB/Simulink application. The IMU system were connected via *I*^2^*C* with Baud rate set to 9600 bits per second; these sensors streamed the 9-axis measurements of accelerometer, gyroscope and magnetometer values (100 Hz) to the development board (Pycom, Wipy 3.0, London, UK) placed on the participants' shoulder. Data from the sensors were merged with the nine degrees of freedom fusion mode (NDOF) of the Bosch sensor and extracted in form of quaternions, acquired by the aforementioned dedicated board (Pycom, Wipy 3.0, London, UK), and streamed to the control system board via UDP to estimate elbow angular speeds and orientations.

**Figure 3:**
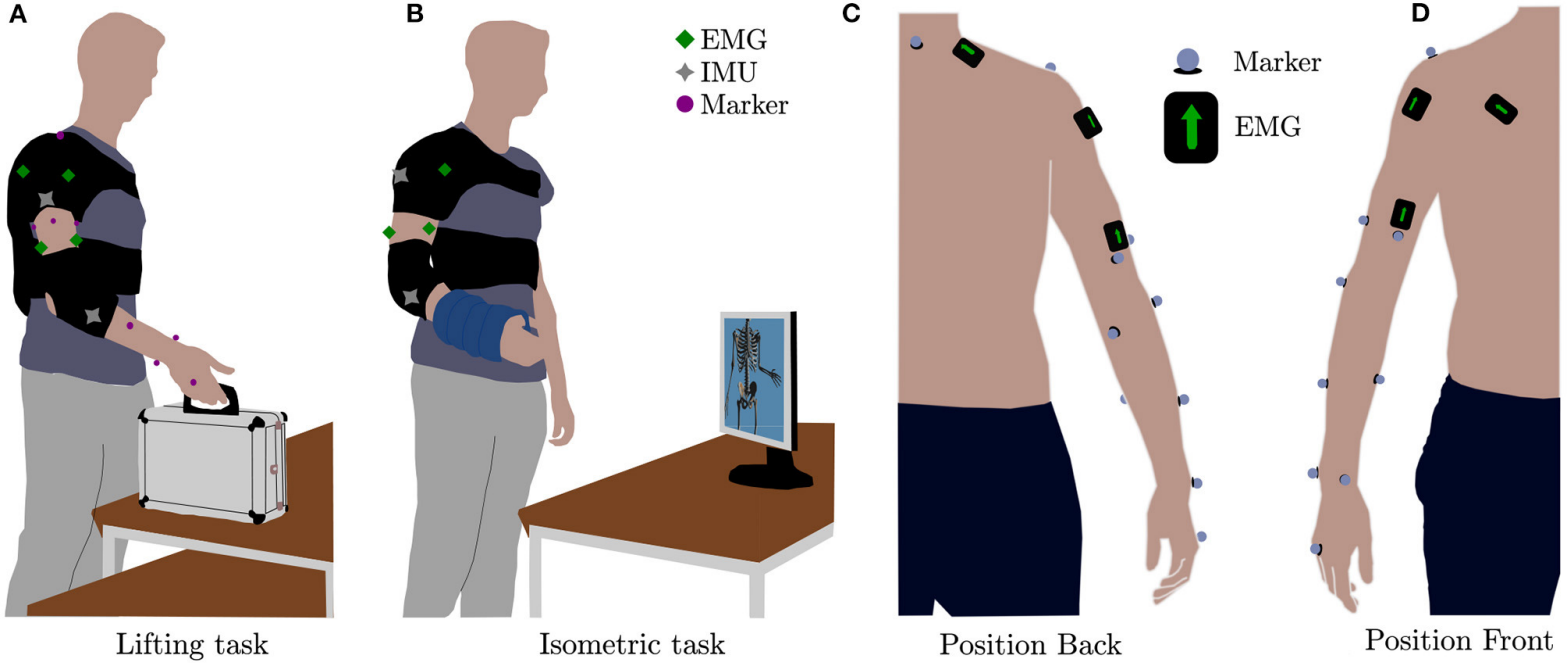
Experimental tasks and setup. **(A)** Simulation of daily living activity involving elbow flexion and extension (*Lifting task*), **(B)** test to study the arise of the fatigue (*Isometric task*), **(C)** position of the electrodes and the markers along the coronal plane (back), and **(D)** position of the electrodes and the markers along the coronal plane (front).

Human kinematics were estimated from recordings of the positions of 12 reflective markers, placed on the participants as shown on Figure 3C (*third metacarpus, lateral wrist, medial wrist, lower arm front, lower arm back, lateral elbow, medial elbow, upper arm front, upper arm back, upper arm side, acromion and c7*) by using a stereophotogrammetric system (Qualisys 5+, Qualisys AB, Göteborg, Sweden).

#### 2.3.1. Experiments

Ten right-handed healthy subjects were recruited for our study (three females, age 27.7 ± 3.6 years, mean ± sd, body weight 78.5 ± 11.6 kg and height 1.80 ± 0.09 m), with no evidence or known history of musculoskeletal or neurological diseases; all participants had normal joint range of motion and muscle strength. They provided explicit written consent to participate in the study. The experimental procedures were carried out in accordance with the Declaration of Helsinki on research involving human subjects and were approved by the Ethical Committee of Heidelberg University (protocol number S-311/2020).

To assess the performance of the device, we designed a protocol in which participants performed two tasks (see Figure 3 and Supplementary Video 1), each in four different assistance conditions: one condition was performed without wearing the exosuit (*no exosuit*, NE) and the others three by changing the *assistance gain* value of the exosuit (Figure 2, orange block) which acts as scaling factor for the exosuit output elbow torque τ_*r*_(*t*) computed by the high-level controller (the myoprocessor). It is worth highlighting that modulation of the *assistance gain* does not change the transient and steady state behavior of the low level admittance controller, but rather tunes the level of assistance by modulating the required control effort at the input of the admittance module. In particular, we chose as assistance levels, 60% (*low assistance* condition, LA), 90% (*medium assistance* condition, MA), and 120% (*high assistance* condition, HA) of the net reference torque τ_*r*_ estimated by the *myoprocessor*.

In the *no exosuit* condition, the IMUs were mounted directly on the subject's arm using velcro straps while in the other three conditions, they were embedded directly in the exosuit harness. We split the protocol in two separate sessions on different days: on the first day we calibrated the high-level controller and carried out the *Lifting task* for all subjects. On the second day, participants performed the *Isometric task* as explained in details in the next sections. The order of the different conditions was semi-randomized across all the subjects, to average out potential order effects (e.g., learning or fatigue).

##### 2.3.1.1. Lifting Task

The aim of the experiment was to assess the exosuit performance, measured by activity of the main muscles involved in the movement of the elbow and shoulder joints. The *Lifting task* (Figure 3A) required the participant to lift a small suitcase with a mass of approximately 2% of the subject's weight. Starting an from an initial position, participants we requested to place the object to a target 20 cm above the initial point. The starting point was set to be the position of the hand when the shoulder was relaxed and the elbow was fully extended along the side of the body.

Verbal cues were given to start each movement: participants were requested to pick the suitcase and placed it to the target location, then wait for the next audio cue to place it back to the initial point. The procedure was repeated five times. While switching between the different conditions (NE, LA, MA, and HA) the subject rested for 5 min to avoid muscular fatigue artifacts.

##### 2.3.1.2. Isometric Task

The isometric task (Figure 3B), was similar the one proposed in (Xiloyannis et al., 2019), and it was designed to assess the impact of the exosuit on the muscular fatigue. The task comprised of a series of five trials where participants held the elbow at 90° for 40 s, separated by 20 s of rest. The task was performed under load conditions wearing two wrist weight bands (1.5 kg each, Reebok, Bolton, United Kingdom). We introduced a resting phase between the different conditions (NE, LA, MA, and HA) of 10 min to avoid muscular fatigue artifacts.

#### 2.3.2. Data Analysis

EMG signals were the online inputs to the myoprocessor module (i.e., biceps brachii and triceps brachii). These were real-time processed through a high-pass filtering (35 Hz) with a second-order Butterworth filter, full wave rectification and a low-pass filtering (4 Hz, second-order Butterworth filter) and normalized to each participant's MVC.

##### 2.3.2.1. Lifting Task

Analysis of the muscular activity was performed by comparing the traces from six muscles in the phases of flexion and extension; raw EMG signals were processed offline to evaluate their root mean square (RMS) as index of activation level across trials and conditions.

We evaluated the flexion and extension phases of movement, where the trajectories segmentation was done by filtering the elbow angle with a Savitzky-Golay filter and setting a threshold (10% of its peak magnitude) on its first time-derivative. We also quantified the effect of robotic support on movement smoothness by looking at the SPectral ARClength (SPARC) index. As mentioned in Balasubramanian et al. (2015), this value is obtained by computing the Fourier magnitude spectrum's arc length of the profile speed.

##### 2.3.2.2. Isometric Task

In order to evaluate muscular fatigue, raw EMG signals were filtered between 15 and 450 Hz with a fourth order Butterworth filter; we used the median frequency (MNF) of the EMG power spectrum and the average rectified value (ARV) in the time domain as indexes of fatigue (Merletti and Parker, 2004), evaluated on eight 3 s epochs during the last isometric contraction. We quantified fatigue as the rate of change of the MNF and ARV, computed by fitting a first order model with a least square method. A steeper positive slope for the ARV and a steeper negative one for the MNF indicated a faster onset of fatigue.

We investigated, by means of the inverse dynamics tool of the OpenSim model, the joint torque at elbow level to assess the effective reduction in the biological torque provided by the exosuit, for increasing levels of assistance. Biological torque was calculated as the difference between the torque estimated from the inverse dynamic model of the arm and the assitive torque provided by the robot.

#### 2.3.3. Statistical Analysis

Performance indexes were compared across conditions with a repeated-measures ANOVA (rANOVA) within-subject factors; the analysis was performed with MiniTab. We considered the four conditions (NE, LA, MA, and HA), the six muscles involved, and their mutual interaction for the *Lifting task* in the phases of flexion and extension.

In the *Isometric task*, we used the same procedure to assert if there were statistical differences between the four conditions (LA, MA, HA, and NE) evaluated in every muscle in the *Detection of muscular fatigue*. We used a rANOVA for the analysis of kinematics, to discriminate any statistical difference in the SPARC index and in the mean elbow angular velocity.

Departure from normality was verified using the Anderson-Darling test and the sphericity condition for rANOVA was assessed with the Mauchly test. When the sphericity condition was violated, we applied the Greenhouse-Geisser correction. When needed, we used *post-hoc* analysis applying the Fisher's LSD test; we took the decision according to the distribution of our data.

Statistical results for ANOVA follow the standard notation *F*(*n, d*), where n are the DOFs of the numerator (i.e., NE, LA, MA, and HA) and d of the denominator (i.e., subjects). The significance level was set at *p* < 0.05; *p*-values below 0.0005 are reported as *p* < 0.000.

## 3. Results

### 3.1. Kinematic Analysis

**Figure 4:**
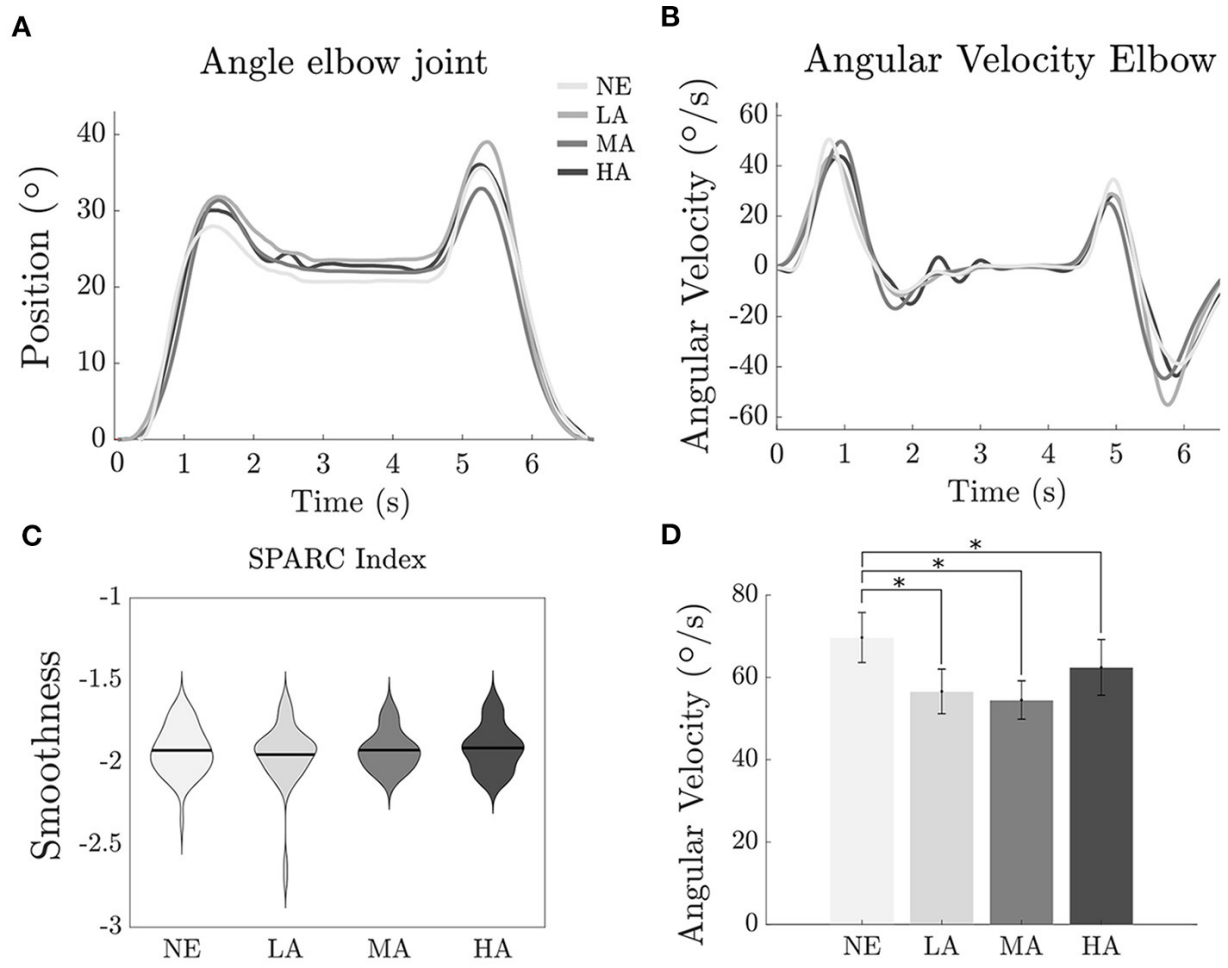
Lifting task: effect of assistance magnitude on human kinematics. **(A)** Angle at the elbow joint along time of one representative subject. **(B)** Angular velocity at the elbow joint along time of one representative subject. **(C)** Violin plot (mean ± sd) SPARC index recorded for the subjects. **(D)** Bar plot of the *Angular Velocity*. *Statistically significant.

The efficacy of an assistive device must be observed with both kinematic and dynamic perspectives: a reduction in muscular activation is acceptable when complemented by a negligible modification of physiological motion during the use of the exosuit. Figures 4A,B show the elbow trajectories for a typical subject during the *Lifting task*, across different levels of assistance. It is clear that different intervention levels of the exosuit do not dramatically change the kinematics of motion, showing that the controller effectively makes both the load and the hardware transparent to the user. In order to demonstrate the same result at the population level, we evaluated movement smoothness across trials and subjects, by looking at the SPARC index (Balasubramanian et al., 2015).

As shown in Figure 4C the index values for the *Lifting task* are very similar between all the conditions; −1.93 ± 0.05, −1.95 ± 0.06, −1.92 ± 0.04, and −1.91 ± 0.05 (mean ± SE), respectively, for NE, LA, MA, and HA, with no significant difference between the four conditions.

Yet, an analysis of the angular velocity at the elbow level showed a slight reduction while the subjects were wearing the exosuit [*F*_(3, 9)_ = 7.69, *p* < 0.000]. We recorded a value of 68.66 ± 6.10° s-1 (mean ± SE) for the NE condition, the values for the LA, MA, and HA were 55.40 ± 5.40, 53.8 ± 4.66, and 60.41 ± 6.78° s-1. Significant differences have been found (*p* < 0.000) between NE and LA, (*p* < 0.000) between NE and MA and (*p* = 0.038) between NE and HA.

### 3.2. Estimation of Muscular Activity

**Figure 5:**
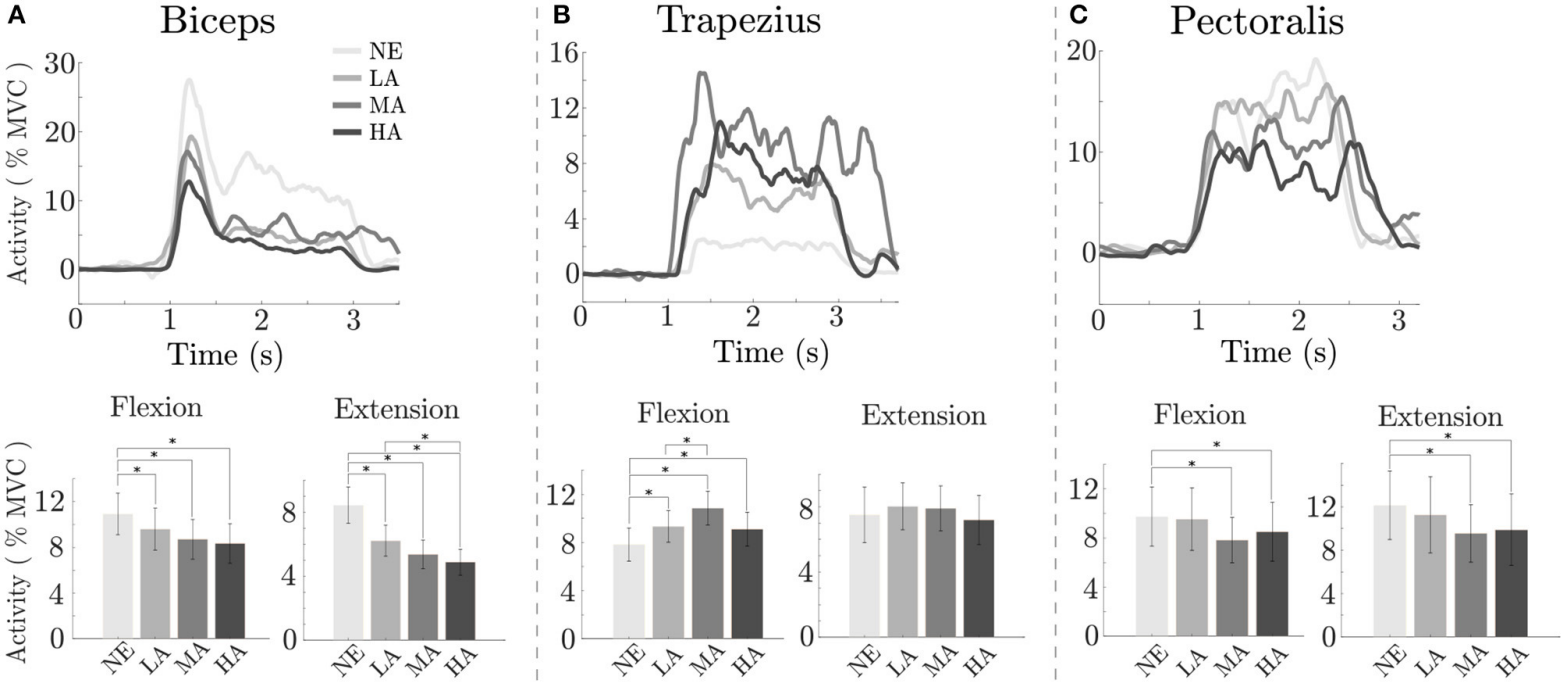
Lifting task: effect of assistance magnitude on muscular activation. On top, envelope of the muscle activity of the biceps brachii, trapezius, and pectoralis major along time of one representative subject (from **A–C**) for the different conditions; on the bottom the averaged RMS, across all subjects, recorded in the phases of *Flexion* and *Extension* relative to the muscle on top. *Statistically significant.

Analysis of muscular activity during the *Lifting task* is depicted on Figure 5: a significant reduction was found in the activity of the main muscle involved during elbow flexion in the sagittal plane (i.e., biceps brachii), present in all assisted conditions when compared to the NE condition, for both the flexion [*F*_(3, 9)_ = 4.42 *p* = 0.005] and extension [*F*_(3, 9)_ = 15.28 *p* < 0.000] phase of movement.

The top row of Figure 5 depicts the EMG envelopes for a typical subject in the four tested conditions: the muscular activation for all the involved muscles differs, and resulted to be lowest when the exosuit provides high assistive torque (HA). At the population level, since the biceps was mostly involved in the lifting task, we analyzed its activity in the flexion phase: it resulted to have values ranging from 10.92 ± 1.81% of MVC [mean ± standard error (SE)] without the exosuit (NE), while, respectively, 9.60 ± 1.82, 8.71 ± 1.74, and 8.34 ± 1.73% during the LA, MA, and HA conditions. As we can notice in Figure 5A, there is a progressive reduction of the biceps activity while increasing the assistance level, with significant differences between LA and NE (*p* = 0.021), between MA and NE (*p* = 0.032) and between HA and NE (*p* = 0.01).

The capacity of interpreting user intention by the EMG-driven model based controller is reflected also in the extension phase, in which we found values ranging from 8.44 ± 1.12, 6.21 ± 0.97, 5.35 ± 0.88, and 4.87 ± 0.81% for the conditions NE, LA, MA, and HA, respectively, with significant values (*p* < 0.000) between LA and NE (*p* < 0.000) MA and NE, (*p* < 0.000) HA and NE, and (*p* = 0.02) between LA and HA. The outcomes show that the assistance is smoothly provided along the whole motion without disrupting user gesture during lifting activities; moreover, as previously stated, in the flexion phase we had a progressive reduction of the muscle activity proportional to the assistance level, indicating that the exosuit intervention is in phase with user's motion and does not interfere in the task completion across different assistance levels.

For the trapezius, we found an increase of the activity in the flexion phase [7.82 ± 1.38, 9.34 ± 1.32, 10.85 ± 1.41, and 9.10 ± 1.41% for the NE, LA, MA, and HA conditions, *F*_(3, 9)_ = 6.96, *p* < 0.000] with significant difference (*p* = 0.02) between LA and NE, (*p* < 0.000) between MA and NE, (*p* = 0.008) between HA and NE and (*p* = 0.044) between MA and LA. Muscular activity of the trapezius in the extension phase of movement was constant across conditions.

Lastly, for the pectoralis we detected, in flexion, mean activity levels of 9.75 ± 2.42, 9.53 ± 2.55, 7.83 ± 1.85, 8.50 ± 2.40% for the NE, LA, MA, and HA, respectively, with significant difference [*F*_(3, 9)_ = 3.65, *p* = 0.014]; we found difference (*p* = 0.036) between MA and NE, and (*p* = 0.027) between HA and NE. In extension, muscular levels were 12.14 ± 3.16, 11.26 ± 3.52, 9.54 ± 2.66, and 9.89 ± 3.29% for the NE, LA, MA, and HA conditions, with significant difference [*F*_(3, 9)_ = 4.86, *p* = 0.003]; we found difference (*p* = 0.006) between MA and NE, and (*p* < 0.000) between HA and NE. As shown in Figure 5C, the activity of the pectoralis when we provided assistance resulted comparable to the NE condition in LA, while in MA and HA we have a visible reduction, even if between these last two conditions muscular activity showed a plateau.

### 3.3. Analysis of Biological Torque

**Figure 6:**
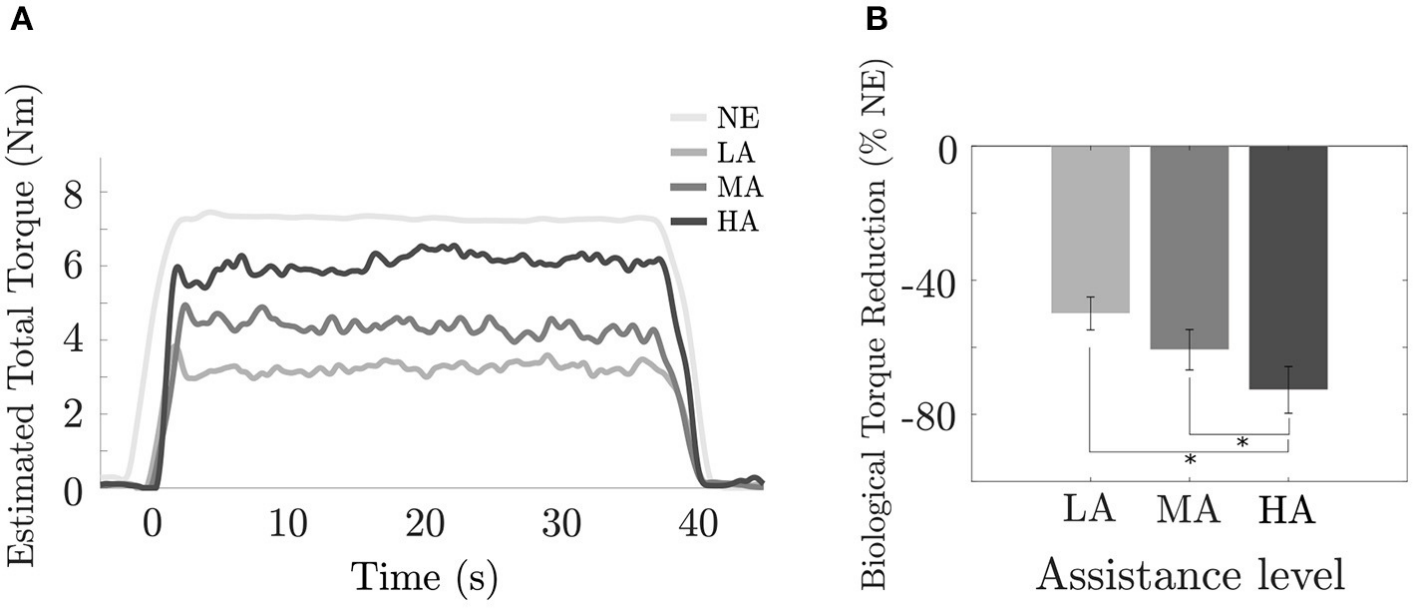
Isometric task: effect of assistance magnitude on exosuit torque. **(A)** Estimated elbow torque for a typical subject during a single trial in NE, LA, MA, and HA conditions; **(B)** Percentage of reduction of subjects muscular effort respect to the unassisted condition (%NE) across different levels of assistance LA, MA, and HA. *Statistically significant.

In Figure 6A, we report the torque at the elbow during the *Isometric task* for a typical subject: the NE condition, where no exosuit was worn and the whole effort came from the user's muscular activity, was evaluated by using her/his scaled Opensim model, while the other curves (LA, MA, HA) were obtained by the torque output τ_*r*_(*t*) from EMG model based controller while delivering assistance.

We found that the biological torque values (Figure 6B) acting on the elbow joint were 7.49 ± 0.48N m (mean ± SE) in the NE condition, while the torque provided by our exosuit was 3.61 ± 0.33N m, 4.39 ± 0.40N m and 5.26 ± 0.46N m for the LA, MA, and HA conditions. A significant reduction (Figure 6B) of the biological torque at the elbow for all the subjects was observed [*F*_(3, 9)_ = 8.66, *p* = 0.001], with a decreasing trend for increasing values of the assistance gain. Assistance from the exosuit resulted in relative changes in the biological torque of −49.86 ± 4.94% (mean ± SE), −60.75 ± 6.03%, and −72.73 ± 6.98% for the LA, MA, and HA conditions, respectively, and a significant difference (*p* = 0.009) between LA and HA and (*p* = 0.038) between MA and HA assistive interventions.

### 3.4. Detection of Muscular Fatigue

In order to study the effect of the assistance on muscular fatigue we used the data recorded during the *Isometric task*, and extracted both ARV and MNF indices, as similarly proposed by Xiloyannis et al. (2019). Here we report the muscles that presented significant difference in their activity during the test.

**Figure 7:**
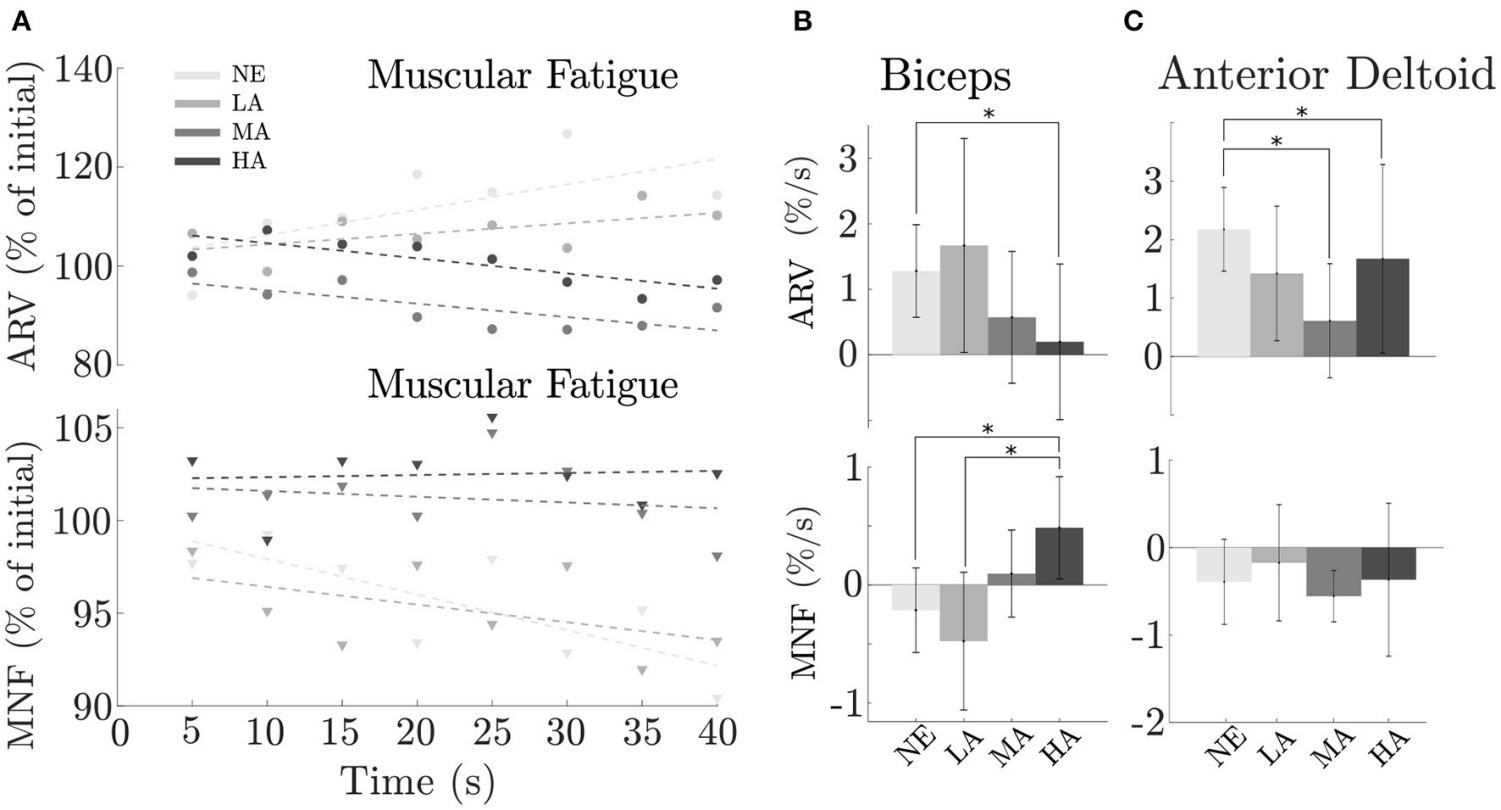
Isometric task: effect of assistance magnitude on muscular fatigue across subjects. **(A)** Biceps brachii AVR (top) and MNF (bottom) values along the eight epochs considered in the last window over time, **(B)** ARV and MNF rate of change averaged between subjects for the biceps (mean ± SE), **(C)** Anterior deltoid AVR and MNF rate of change averaged between subjects (mean ± SE). *Statistically significant.

As shown in Figures 7A,B, changes in assistance level tend to postpone the onset of muscular fatigue for the biceps brachii, which showed a progressive decrease [*F*_(3, 9)_ = 3.33, *p* = 0.035] of the ARV rate of change from the NE (unassisted) condition 1.28 ± 0.67%/s (mean ± SE) gradually passing trough the assistance conditions LA, MA, and HA, in the order 1.67 ± 1.54, 0.57 ± 0.95, and 0.20 ± 1.12%/s. A a significant difference was found (*p* = 0.032) between NE and HA conditions corresponding, respectively, to unassisted motion and maximum assistance level.

Unlike the trend noted in the previous ARV analysis, but confirming the result of postponing muscular fatigue, we found a positive progression in the MNF rate of change [*F*_(3, 9)_ = 2.56, *p* = 0.05] across conditions: −0.21 ± 0.34, −0.48 ± 0.55, 0.10 ± 0.35, and 0.41 ± 0.43%/s in the NE, LA, MA, and HA, respectively, with a significant difference (*p* = 0.031) between HA and NE and (*p* = 0.019) between HA and LA.

Although the major effort in task execution was attributed to the biceps, it is worth considering the other main agonist muscle involved in the *Isometric task*: the anterior deltoid. Results did not clearly show a relation between muscular fatigue and assistance level, probably because the anterior deltoid was not directly supported by the exosuit. As depicted in Figure 7C, we obtained an AVR value [*F*_(3, 9)_ = 2.47, *p* = 0.05] of 2.18 ± 0.72, 2.40 ± 1.15, 0.61 ± 0.98, and 1.67 ± 1.61%/s (mean ± SE) in NE, LA, MA, and HA conditions, respectively, with significant difference (*p* = 0.047) between MA and NE and (*p* = 0.05) between HA and NE.

We found a rate of change in the MNF with the following values across conditions −0.52 ± 0.49%/s (NE), −0.12 ± 0.66%/s (LA), −0.64 ± 0.29%/s (MA), and −0.44 ± 0.87%/s (HA). Yet, also for such indicator, there was no clear trend on how assistance level may affect muscular fatigue.

## 4. Discussion

Understanding the mechanisms underlying the impact of assistive technology on the natural biomechanics is the key aspect to design a reliable hardware which can feature a synergistic coexistence with the user and can be fully accepted in daily life. The initial and most accepted hypothesis, “stronger is better,” respectively, related to the power from the device and the resulting consequent human performance, is somehow disrupted if we rethink and assume that both the hardware and its biological counterpart are not linearly interconnected: therefore the mechanism underlying their mutual matching depends on multiple factors opportunely shaped to fit. On this last aspect a clarifying classical example in automotive might shed light: it is useless to mount a heavy powerful engine on a frame designed to be agile and light. The result would inevitable mismatch, annihilating the single strengths of the two main components. Generalizing the aforementioned example, the main target of our study was to experimentally isolate and characterize the relationship between the level of assistive intervention from the machine and the response in terms of muscular activation from the user. For an exosuit or a wearable device in general, the mechatronics must be able to closely feel the wearer's nature and facilitates the mutual interaction, promoting on the human side the rise of a motor adaptation and at last a symbiosis between the two artificial and biological entities. As previously demonstrated on Lotti et al. (2020), the myoprocessor approach is a suitable way to directly interface the human motion intention and assistance needed to the exosuit control. The new control framework has been simplified by keeping only the main agonist and antagonist elbow flexion muscles (i.e., long head of thee triceps and long head of the triceps). It is important to remark that we need to include at least one antagonist to not avoid the passive elastic muscle force in the joint torque estimation, as discussed also in Lotti et al. (2020). The proposed controller and the associated hardware, demonstrated that an increasing level of assistance is mastered by the user, without needing long familiarization to adapt to the device, resulting in a dynamic and kinematic matching intuitively reached and almost instantaneous.

In the next section we will discuss in details all the multiple aspects arisen from the outcomes herewith presented and, we will discuss extensively not only about the controller performance but also on the importance of the ergonomics in coupling the human and the hardware.

### 4.1. Increasing Assistance Magnitude Results in Increasing Muscular Benefit

Our first hypothesis was that increasing the assistance of the myoelectric controller will lead to an increasingly higher reduction in the activation of the major muscles working in parallel with the exosuit. As shown in the *Estimation of muscular activity* (section 3.2), the device driven by the *myoprocessor* is able to reduce the muscular activity of the biceps and the pectoralis without causing unwanted behavior and perceptively altering the natural motion (i.e., lifting task). In particular looking at the biceps (Figure 5A, bottom), the most responsible muscle for task execution, a progressive activity reduction has been noticed and it demonstrated to be directly related to the assistance levels delivered by the exosuit. Surprisingly, the study revealed that also other muscles, not directly supported by the device, gained benefits in terms of activation as function of the assistance. Indeed, the reduction registered in the pectoralis can depend on two main components. The first component might be related to the passive action of the harness of the exosuit, designed to keep the shoulder in a physiological position, correcting its posture and partly releasing the dynamics of the task on the corresponding joint.

The second one can be seen as a consequence of the biceps activity reduction and it can be linked to the active assistance of the exosuit which, reducing the biological torque at the elbow (Figure 6B) consequently decreases the stress on the whole upper limb avoiding co-contraction or additional synergistic activation from other muscles.

It is hard to predict the trend of muscular activation if we were to investigate higher assistive gains; in this study, we were limited by motor speed characteristics and did not investigate magnitudes above 120%. Additional studies that sweep to higher magnitudes of assistance are required to determine whether the descending trend in muscular activation of the biceps brachii continues.

On the other side, sub-optimal ergonomics of the exosuit might also play a negative role, by increasing the activity of antagonist muscles, as we observed for the trapezius. This increase of the activity of the trapezius, as the assistance of the exosuit rises, is probably a result of by the parasitic actions of the transmission line made by the Bowden cable connecting the proximal anchor point with the actuator stage. Such component, passing on the trapezius before attaching the first anchor point on the shoulder, increases its stiffness when the cable is in tension during assisted elbow flexion. As consequence, the Bowden cover was slightly pressing down the shoulder forcing the subjects to react and contract the trapezius during task execution. Figure 5B clearly illustrate such effect showing an increase of the muscular activity for all the assistance conditions (LA, MA, and HA) with a peak in the MA one. The described issue marks the importance of a coordinated design approach which links a robust control implementation to a tailored ergonomics. By changing the shape of the anchor points to redistribute the pressure, or routing the Bowden cable sheath in such a way to reduce unwanted activity, are some of the basic design specifications to provide an optimized match of the exosuit.

### 4.2. Exosuit Assistance Does Not Affect Movement Smoothness

The synergistic matching between the exosuit and the user has been achieved by combining an accurate and personalized musculoskeletal model with the *myoprocessor*. This has been confirmed by the analysis reported in the results where fatigue onset was delayed when assistance level was increased: a similar finding is described in Park and Cho (2017), where a cable-driven suit for the shoulder reduced the fatigue in the anterior and medial deltoid on five healthy participants. Unfortunately, a quantitative comparison between the study is not possible because of the different metrics used to assess fatigue.

Yet, as previously introduced in the discussions, effectiveness of a wearable device is a multi-faced coin, where not only dynamic aspects (fatigue and muscular activities) but also kinematic effects are depicted. The exosuit must provide support and assistance moving synchronously with the user and doing this means to match the biomechanical bandwidths of a human limb in both force and speed: considering the current available technology, natural actuation and specifically biological muscles are still unmatched.

However, control and mechatronics solutions allow to partly cover the wide range of kineto-dynamic bandwidths of human motion, if one finds the right combination and tuning between online biosignals processing and actuation. In our contribution we try to find the right balance between an effective range of assistance and kinematic performance, in order to cover, at least in part and for specific tasks, user's capabilities. The kinematic effects arising from wearing the exosuit prove that different assistance levels do not alter natural movements smoothness: our results showed that the SPARC index remained constant in all the conditions meaning that the suit moves almost synchronously with the wearer without hindering the movement. This is an extremely crucial aspect in wearable technology, where perceivable minimal delays between the actuation and the user voluntary motion completely disrupt synergistic movements leading to unnatural behaviors, which require an extensive motor adaptation and a demanding cognitive load before accepting/mastering the device (Flemisch et al., 2012). This is an aspect extensively studied in prosthetics where an unmatched dynamics between the human and mechatronic actors often lead to an absence of sense of agency and control authority. We demonstrated that a high level of control authority is reached (in part) with the inclusion of the muscle activity in the loop, that not only reflects the user's motion intention, but triggers the control in a robust fashion by predicting and allowing an almost natural motion.

However, analyzing more in the detail the movements performed by the participants during the *Lifting task* we found (Figure 4B), a significant reduction of the angular velocity at the elbow between the no assisted (NE) and the other three assisted conditions (LA, MA, and HA), highlighting that, although minimally, the exosuit is not perceived as totally transparent. This aspect is extensively discussed by Desmurget et al. (1997) where the authors stated that movements constrained by contact with an external body involve a fundamentally different control strategy from unconstrained conditions, which can affect their duration.

Furthermore, looking at the trend in the assistance magnitude we can notice that increasing the assistive torque leads to higher speed of the elbow (Figure 4D) which tends to resemble the natural speed in the unassisted condition. The reason of such trend can be identified with technological limits of the device mainly due to non-linear phenomena which play a role at low speed: static friction and backlash in the Bowden cables and deformation and migration of the fabric with slack in the tendons, introduce latency and affect the transient behavior of the controller, especially for low speeds.

There are limitations in the study: the exosuit prototype was tested only in a range of assistance which ensures stability and passivity of the device and we should have tested more assistance levels to precisely address the plateauing, and more accurately describe the relation between assistance magnitude and response. We enrolled only a limited cohort of healthy participants, while for the final target the technology should explore the performance on a realistic scenario. We will take into account also the participants' feelings to use the device out to the laboratory conditions through a usability questionnaire. Finally, we want to explore the performances in presence of limited arm mobility and muscular activity (e.g., neuromuscular diseases or motor impairments).

### 4.3. Final Remarks

With the present study we investigated the effects of assistance magnitude on human performance. The outcomes have clearly highlighted that a myoelectric-driven exosuit can lead to consistent advantages in terms of muscular activation magnitude and delay in the onset of fatigue, for increasing assistive levels.

Due to the inclusion of the muscular activity in the control loop, we also established that the increase of assistive magnitude in the *myoprocessor* does not lead to a decrease of movement smoothness.

Our results suggested that the combination of the proposed hardware and control frameworks is reliable in terms of movement intention detection and real-time assistance adaptation, and if opportunely engineered it would be able to embrace a wide range of applications in assistive technology.

## Data Availability Statement

The raw data supporting the conclusions of this article will be made available by the authors, without undue reservation.

## Ethics Statement

The studies involving human participants were reviewed and approved by Universität Heidelberg Ethikkommission der Med. Fakultät. The patients/participants provided their written informed consent to participate in this study. Written informed consent was obtained from the individual(s) for the publication of any potentially identifiable images or data included in this article.

## Author Contributions

FM, NL, and LM conceived the new exosuits. NL, LM, and FM implemented the controller. FM, NL, and LS designed and performed the experiments. MX and RR helped drafting the paper. FM, NL, LS, and MX analyzed and interpreted the data. All authors provided critical feedback on the manuscript. All authors read and approved the final manuscript.

## Conflict of Interest

The authors declare that the research was conducted in the absence of any commercial or financial relationships that could be construed as a potential conflict of interest.
